# Tryptophan promotes charitable donating

**DOI:** 10.3389/fpsyg.2014.01451

**Published:** 2014-12-17

**Authors:** Laura Steenbergen, Roberta Sellaro, Lorenza S. Colzato

**Affiliations:** Cognitive Psychology Unit, Institute for Psychological Research and Leiden Institute for Brain and Cognition, Leiden UniversityLeiden, Netherlands

**Keywords:** tryptophan, serotonin, charity

## Abstract

The link between serotonin (5-HT) and one of the most important elements of prosocial behavior, charity, has remained largely uninvestigated. In the present study, we tested whether charitable donating can be promoted by administering the food supplement L-Tryptophan (TRP), the biochemical precursor of 5-HT. Participants were compared with respect to the amount of money they donated when given the opportunity to make a charitable donation. As expected, compared to a neutral placebo, TRP appears to increase the participants’ willingness to donate money to a charity. This result supports the idea that the food we eat may act as a cognitive enhancer modulating the way we think and perceive the world and others.

## INTRODUCTION

“Every good act is charity. A man’s true wealth hereafter is the good that he does in this world to his fellow”, Molière once said. Indeed, charitable donating is an essential component of prosocial behavior and a key determinant of social reliability (Milinski et al., 2002).

Pharmacological studies in rats and humans suggest that the neurotransmitter serotonin (5-HT) plays a crucial role in promoting prosocial behavior (Crockett, 2009). Indeed, as pointed out by Siegel and Crockett (2013), serotonergic levels tend to be negatively correlated with antisocial behaviors such as social isolation and aggression, and tend to be positively correlated with prosocial behaviors such as cooperation and affiliation. Prosocial behavior can be reduced by lowering 5-HT levels through tryptophan depletion (Wood et al., 2006; Crockett et al., 2008, 2009) and enhanced through administering the food supplement L-Tryptophan (TRP), the biochemical precursor of 5-HT (Colzato et al., 2013) or through administering selective serotonin reuptake inhibitors (Knutson et al., 1998; Tse and Bond, 2002).

Here, for the first time, we investigated whether the administration of the essential amino acid TRP, contained in food such as fish, eggs, soy, and milk, can promote charitable donating. TRP supplementation is known to increase plasma TRP levels and to influence brain 5-HT synthesis (Markus et al., 2008). We expected to find a beneficial effect of TRP on charitable donating because donating was found to selectively activate the subgenual–septal area (Moll et al., 2006), which shares reciprocal anatomical connections with raphe nuclei (Drevets, 2001), the principal release center of 5-HT in the brain. Hence, it is likely that the activation of the subgenual–septal area is modulated through serotonergic projections—which we aimed to target by the supplementation of TRP.

## MATERIALS AND METHODS

### PARTICIPANTS

Thirty-two healthy international south European students (mean age = 21.8; 4 male, 28 female; mean Body Mass Index = 21.5, range 17.8–30.8) with no cardiac, hepatic, renal, neurological, or psychiatric disorders, personal or family history of depression, migraine and medication or drug use participated in the experiment. Participants were screened via a phone call by the experiment leader before inclusion, using the mini international neuropsychiatric interview (MINI; Sheehan et al., 1998). The MINI is a short, structured, interview of about 15 minutes that screens for several psychiatric disorders and drug use, often used in clinical and pharmacological research (Sheehan et al., 1998; Colzato and Hommel, 2008; Colzato et al., 2009). Participants were randomly assigned to two experimental groups. Sixteen participants (2 male, 14 female) were exposed to an oral dose (powder) of 0.8 grams of TRP (supplied by AOV International Ltd.)—which roughly corresponds to the amount of TRP contained in 3 eggs–and 16 (2 male, 14 female) to 0.8 grams of microcrystalline cellulose (Sigma-Aldrich Co. LLC), a neutral placebo. These doses were always dissolved in 200 ml of orange juice. Following Markus et al. (2008) and Colzato et al. (2013) women using contraception were tested when they actually used the contraception pill. On each experimental morning, participants arrived at the laboratory at 9:30 am. Participants had been instructed to fast overnight (eating was not allowed after 11:00 pm); only water or tea without sugar was permitted. In addition, subjects were not allowed to use any kind of drugs before or during the experiment, or to drink alcohol from the day before their participation until their completion of the study.

Written informed consent was obtained from all subjects; the protocol and the remuneration arrangements of 10 Euros were approved by the local ethical committee (Leiden University, Institute for Psychological Research).

### PROCEDURE

All participants were tested individually. Upon arrival, following Colzato et al. (2013), participants were asked to rate their mood on a 9 × 9 Pleasure × Arousal grid (Russell et al., 1989) with values ranging from –4 to 4. Heart rate (HR; in beats per minute) and systolic and diastolic blood pressure (SBP and DBP) were collected from the non-dominant arm with an OSZ 3 Automatic Digital Electronic Wrist Blood Pressure Monitor (Spiedel & Keller). One hour following the administration of TRP (corresponding to the beginning of the 1 hour-peak of the plasma concentration; Markus et al., 2008) or placebo, participants again rated their mood, before having HR, SBP, and DBP measured for the second time.

Next, after having performed an unrelated computer-based attentional blink task that requires the detection of two targets in a rapid visual on-screen presentation, which took about 30 minutes, participants were presented with the donating task. After the donating task, participants again rated their mood, before having HR, SBP, and DBP measured for the third time.

### DONATING TASK

Following van IJzendoorn et al. (2011), participants were not informed beforehand that the donating task was part of the experiment. Donating behavior was measured by the amount of money the participant donated. After having received 10 Euros (one 5-Euronote, two 1-Euro coins, and 6 Fifty-cent coins) for their participation in the study, participants were left alone and asked whether they were willing to donate part of their financial reward to charity. Four money boxes (Unicef, Amnesty International, Greenpeace, and World Wildlife Fund) had been positioned on the table. All money boxes were filled with four Fifty-cent coins in order to enhance credibility (see van IJzendoorn et al., 2010, 2011, for a similar task).

Hence, the donating task was standardized, without the presence of an experimenter, and with a fixed amount of money in a fixed number of notes and coins. The donating task used in the van IJzendoorn et al. (2011) study was similar in terms of participants donating their own money to a real charity but it differed in terms of having the choice between four different charities compared to solely Unicef. Donated money was transferred to the bank accounts of the charities after data collection.

### STATISTICAL ANALYSIS

Heart rate, systolic and diastolic blood pressure, mood, and arousal were analyzed separately by means of repeated-measures analyses of variance (ANOVAs) with effect of time (first vs. second vs. third measurement) as within-subjects factor and with group (Placebo vs. TRP) as between-group factor. A *t*-test for independent groups was performed to assess differences between the two groups (Placebo vs. TRP) in the amount of money donated. Effect magnitudes were assessed by calculating Cohen’s d (Cohen, 1988). A significance level of *p* < 0.05 was adopted for all statistical tests.

## RESULTS

### PARTICIPANTS

No significant differences were found among group with respect to age (21.7, SD = 1.4 vs. 21.8, SD = 2.9, for the placebo and TRP group respectively), *t*(30) = 0.15, *p* > 0. 88, and sex, χ2 (1, *N* = 32) = 0.00, *p* = 1.00.

### DONATING TASK

As expected, participants donated significantly more euros to the charities in the TRP condition (1.00, SD = 0.79) than in the placebo condition (0.47, SD = 0.59), *t*(30) = 2.14, *p* < 0.05, Cohen’s δ = 0.78.

### PHYSIOLOGICAL AND MOOD MEASUREMENTS

Analysis of variance s revealed that HR (78, SD = 10.9 vs. 72, SD = 9.4 vs. 69, SD = 9.2 and 75, SD = 13.1 vs. 71, SD = 8.5 vs. 68, SD = 11.2, after placebo and TRP, respectively), DBP (68, SD = 10.4 vs. 63, SD = 9.0 vs. 64, SD = 10.6 and 71, SD = 8.5 vs. 66, SD = 7.2 vs. 70, SD = 9.4, after placebo and TRP), SBP (110, SD = 12.8 vs. 104, SD = 11.2 vs. 106, SD = 11.8 and 114, SD = 8.1 vs. 109, SD = 10.3 vs. 112, SD = 9.7, after placebo and TRP), mood (0.9, SD = 1.4 vs. 1.4, SD = 1.4 vs. 1.2, SD = 1.4 and 0.9, SD = 1.6 vs. 1.0, SD = 1.6 vs. 1.2, SD = 1.6, after placebo and TRP), and arousal (0.6, SD = 1.8 vs. 1.1, SD = 1.5 vs. 0.4, SD = 1.8 and 0.5, SD = 1.6 vs. 0.5, SD = 1.5 vs. 0.2, SD = 1.3, after placebo and TRP) did not significantly change after the intake of TRP, *F*’*s* < 1.

## DISCUSSION

The present study is the first demonstration that charitable donating can be enhanced by serotonin-related food supplements. We argued that TRP supplementation, and the resulting boost in 5-HT should be beneficial for the participants’ willingness to donate money to a charity.

One may wonder how this novel finding relates to the observation of Barraza et al. (2011) and van IJzendoorn et al. (2011) that the neuropeptide oxytocin (OT) also increases charitable donation. Serotonergic terminals, mainly originating from the dorsal and median raphe nuclei of the brainstem, project to the paraventricular nuclei (Larsen et al., 1996), where the neurons release OT. So, comparable effects on prosocial behavior of TRP and OT are conceivable if one considers the functional and anatomical interactions between serotonergic and oxytocinergic systems. Further, the administration of the serotonergic agonist fenfluramine to healthy subjects increases plasma OT levels (Lee et al., 2003). Thus, it may be likely that the willingness to donate money to a charity is modulated by the effect that 5-HT exerts on OT levels.

More research is needed to extend and replicate our preliminary findings with a bigger sample size. Follow-up studies should correlate the amount of money donated with plasma levels of TRP. Finally, to evaluate the effect of the TRP administration on the brain, it would be interesting to investigate whether TRP supplementation is associated with increased blood oxygenation level dependent (BOLD) changes in the fronto-mesolimbic networks, which are associated with charitable donating (Moll et al., 2006).

The present study is the first to show that TRP promotes charitable donating, an important element of prosocial behavior. Our results support the materialist approach that “you are what you eat” (Feuerbach, 1960)—the idea that the food one eats has a bearing on one’s state of mind. The food we eat may thus act as a cognitive enhancer that modulates the way we deal with the “social” world. In particular, the supplementation of TRP, or TRP-containing diets, may support the prosocial behavior of charity that Molière was concerned about.

## Conflict of Interest Statement

The authors declare that the research was conducted in the absence of any commercial or financial relationships that could be construed as a potential conflict of interest.
